# Policy dimensions of global wastewater surveillance

**DOI:** 10.2471/BLT.24.292245

**Published:** 2024-09-01

**Authors:** Megan B Diamond, Toni Whistler, Karina Rando, Chioma Nwachukwu, Mukhlid Yousif

**Affiliations:** a The Rockefeller Foundation, 52 Clark St Apt 8R, Brooklyn, New York, 11201, United States of America.; b The Global Fund to Fight AIDS, Tuberculosis and Malaria, Geneva, Switzerland.; c Ministry of Health, Montevideo, Uruguay.; d Gavi, the Vaccine Alliance, Geneva, Switzerland.; e Center for Vaccines and Immunology, National Institute for Communicable Diseases, Johannesburg, South Africa.

Wastewater and environmental surveillance gained global attention during the coronavirus disease 2019 (COVID-19) pandemic. Confronted with a scarcity of diagnostic data, public health authorities, academics and research institutions turned to analysing sewage to understand the changing burden of severe acute respiratory syndrome coronavirus 2 (SARS-CoV-2) in communities.^1^ Today, over 70 countries worldwide use wastewater surveillance tools in some capacity.^2^^,^^3^ However, the underlying policy frameworks supporting wastewater surveillance remain weak, as governments constructed them in a time of crisis through ad hoc mechanisms and practical collaborations. As the world transitions towards other global health priorities and prepares for the next pandemic, progress towards scaling up wastewater surveillance should not be lost.

SARS-CoV-2 and poliovirus represent the strongest evidence base for the successful use of wastewater surveillance to inform public health action. However, both cases are disease-specific and were enabled by unique policy contexts. As the science and demand around wastewater surveillance grow beyond individual pathogens, so too must global policies.

Here, we outline a two-pronged, policy-based approach for the sustainable use of multipathogen wastewater surveillance for public health. 

First, enhancing the World Health Organization (WHO) capacity in wastewater surveillance is a prerequisite for the effectiveness of any global wastewater surveillance efforts. WHO has long recognized the role of environmental surveillance in monitoring disease threats, though activities are mainly run within disease-specific vertical programmes rather than across the Organization.^4^ In April 2022, the WHO Water, Sanitation and Hygiene team released the first version of environmental surveillance guidance for SARS-CoV-2.^4^^,^^5^ However, a coordinated and expanded vision for the field has not emerged. The launch of the WHO Hub for Pandemic and Epidemic Intelligence^6^ in 2021 has prepared the Organization to implement horizontal programming that capitalizes on advanced disease-specific activities. WHO leadership could inform Member States of the evolving science of the field; unlock access to normative guidance for collecting, analysing, interpreting and responding to wastewater results; and support integration with other existing data sources. As a trusted convener and knowledge broker, WHO is positioned to facilitate partnerships that enable information sharing to improve global health security. The Organization, through technical guidance, can strengthen representation from low- and middle-income countries where expanded wastewater surveillance efforts have been ongoing, and collaboration with GLOWACON, the Global Consortium for Wastewater and Environmental Surveillance for Public Health,^7^ a critical partner network that addresses challenges to the uptake of wastewater surveillance and supports the European Union Wastewater Observatory for Public Health.^8^

Second, collaboration and coordination among health institutions, industry partners, funding agencies and other stakeholders at a global level are crucial to achieve a joint understanding of the opportunities wastewater surveillance presents. Multiple resources are needed to strengthen wastewater surveillance; fill scientific gaps regarding the feasibility and actionability of keeping different pathogens under surveillance; create digital infrastructure; enable utilization of findings; and strengthen mechanisms to ensure the continuous availability and affordability of consumables, reagents, supplies and dedicated human resources. Additionally, regulatory standards and a common policy framework for country action that sets clear targets and outlines strategies that can be used to turn national policies into practical operational programmes at local level are needed.

Alliances and coalitions have long been critical to address the complex global health challenges that require engagement from different sectors. Gavi, the Vaccine Alliance and the Coalition for Epidemic Preparedness Innovations contribute to public health and therefore can play a key role in wastewater surveillance.^9^^,^^10^ The governance models in these broad coalitions ensure a system-based approach and are designed to support low- and middle-income countries. The partnerships will allow to transition from short-term wastewater surveillance projects to sustainable programmes embedded in public health departments.

## References

[1] Keshaviah A, Diamond MB, Wade MJ, Scarpino SV, Ahmed W, Amman F, et al. Global Wastewater Action Group. Wastewater monitoring can anchor global disease surveillance systems. Lancet Glob Health. 2023 Jun;11(6):e976–81. 10.1016/S2214-109X(23)00170-5 37202030

[2] Naughton CC, Roman FA Jr, Alvarado AGF, Tariqi AQ, Deeming MA, Kadonsky KF, et al. Show us the data: global COVID-19 wastewater monitoring efforts, equity, and gaps. FEMS Microbes. 2023 Jan 12;4:xtad003. 10.1093/femsmc/xtad003 37333436 PMC10117741

[3] COVIDPoops19. Redlands: ArcGIS; 2024. Available from: https://www.arcgis.com/apps/dashboards/c778145ea5bb4daeb58d31afee389082 [cited 2024 Aug 9].

[4] Guidelines for environmental surveillance of poliovirus circulation. Geneva: World Health Organization; 2003. Available from: https://iris.who.int/handle/10665/67854 [2024 Aug 8].

[5] Environmental surveillance for SARS-CoV-2 to complement other public health surveillance. Geneva: World Health Organization; 2023. Available from: https://www.who.int/publications/i/item/9789240080638 [2023 Sep 12].

[6] The WHO Hub for Pandemic and Epidemic Intelligence. Geneva: World Health Organization; 2024. Available from: https://pandemichub.who.int/ [cited 2024 Aug 8].

[7] Gawlik BM, Comero S, Tavazzi S, Maffettone R, Glowacka N, Pierannunzi F, et al. The International Conference “Towards a Global Wastewater Surveillance System for Public Health”. Luxembourg: Publication Office of the European Union; 2024. 10.2760/425638

[8] EU Wastewater Observatory for Public Health. Brussels: European Commission; 2024. Available from: https://wastewater-observatory.jrc.ec.europa.eu/ [2024 Apr 17].

[9] COVAX. Key learnings for future pandemic preparedness and response. Geneva: Gavi, the Vaccine Alliance; 2022. Available from: https://www.gavi.org/sites/default/files/covid/covax/COVAX_Key-Learnings-for-Future.pdf [2024 Aug 9].

[10] Brende B, Farrar J, Gashumba D, Moedas C, Mundel T, Shiozaki Y, et al. CEPI ­– a new global R&D organisation for epidemic preparedness and response. Lancet. 2017 Jan 21;389(10066):233–5. 10.1016/S0140-6736(17)30131-9 28109539 PMC7138390

